# Supplementary material for: miR2118-dependent U-rich phasiRNA production in rice anther wall development
Source: Nat Commun. 2020 Jun 19;11:3115. doi: 10.1038/s41467-020-16637-3 (PMC7305157; doi:10.1038/s41467-020-16637-3)
Supplement: Supplementary file 1 — Supplementary Information [file 41467_2020_16637_MOESM1_ESM.pdf]

Supplementary Information

miR2118-dependent U-rich phasiRNA production in rice anther wall development

Komiya *et al*.

Supplementary Figures 1 to 11
Supplementary Tables 1 to 4

Supplementary Figure 1

a

Table. Identified deletion or/and insertion in seven miR2118 loci of a gene-editing mutant

**miR2118 d**	CCTTGTTTTCCTGATGCCT deletion & CGCCTCTAGAACTAGGGGCTGAGATTTGGTT insertion																							biallelic
**miR2118 f**	T	A	G	G	A	A	T	G	G	G	A	G	G	C	A	T	C	A	G	G	A	A		biallelic
	T	A	G	G	A	A	T	G	G	G	A	G	G	C	A	A	T	C	A	G	G	A	A	
**miR2118 j**	T	A	G	G	A	A	T	G	G	G	A	G	G	C	A	T	C	A	G	G	A	A		monoallelic
**miR2118 k**	T	A	G	G	A	A	T	G	A	G	A	G	G	C	A	A	T	C	A	G	G	A	A	monoallelic
**miR2118 m**	T	A	G	G	A	A	T	G	G	G	A	G	G	C	C	A	T	C	A	G	G	A	A	biallelic
	T	A	G	G	A	A	T	G	G	G	A	G	G	C	A	T	C	A	G	G	A	A		
**miR2118 n**	T	A	G	G	A	A	T	G	G	G	A	G	G	C	A	T	C	G	G	G	A	A		biallelic
	T	A	G	G	A	A	T	G	G	G	A	G	G	C	T	A	T	C	G	G	G	A	A	
**miR2118 p**	T	A	G	G	C	T	T	G	G	G	A	G	G	C	A	T	C	G	G	G	A	A		biallelic
	T	A	G	G	C	A	T	G	G	G	A	G	G	C	A	A	T	C	G	G	G	A	A	
																						T: insertion		deletion

b

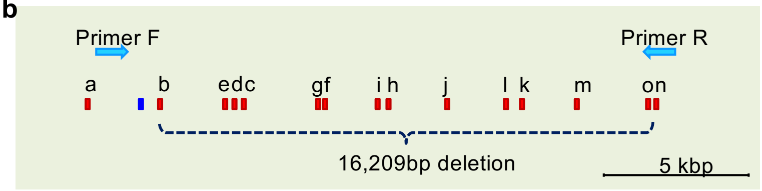

c

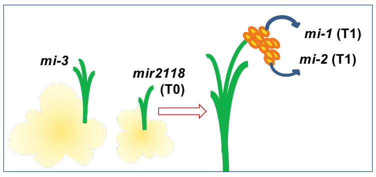

d

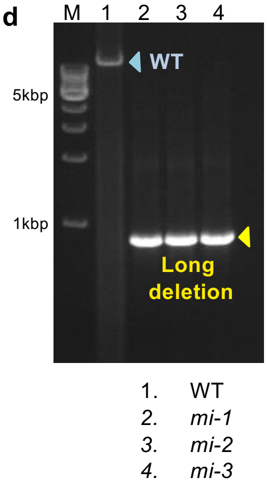

e

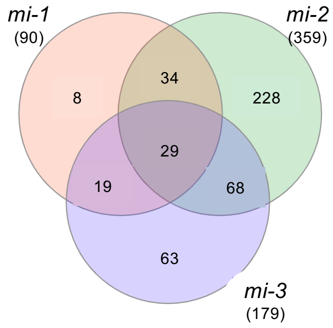

f

Table. Identification genetic variant numbers in *21PHAS* clusters

	1Kbp Up Stream of *21PHAS*	1Kbp Down Stream of *21PHAS*	*21PHAS* (Body)
mi-1 vs NB	0	0	0
mi-2 vs NB	2	4	0
mi-3 vs NB	1	1	0

Supplementary Figure 1. Variation of the *mir2118* mutant lines.
a. Table of identified deletion and/or insertion mutations at seven miR2118 family loci of a gene editing mutant. This mutant (T0) demonstrated complete sterility, and therefore this line was not used for further experiments. **b.** Schematic structure of miR2118 gene clusters (a-n) at chromosome 4. Light blue arrows represent the forward and reverse primers to detect deletions of the miR2118 loci. **c.** Origins of three *mir2118*-deleted lines, *mi-1, mi-2*, and *mi-3*. **d.** PCR analysis detects long deletion in the *mir2118* mutants using the primers shown in **(b). e.** A Venn-diagram for genetic variations identified among *mi-1, mi-2,* and *mi-3*. **f.** Table for identified genetic variations in the 21*PHAS* body at 1kb upstream and downstream in the clusters of three *mir2118* lines. NB means "Nipponbare" genome used as control.

Supplementary Figure 2

a

Table. Segregation rate of ***mir2118*** in BCF2 population				
	**WT/H (+/+; +/-)**	**homozygous (-/-)**	**X-squared**	***p*-value**
mi-1	48	20	0.70588	0.4008
mi-2	41	9	1.3067	0.253
mi-3	35	8	0.93798	0.3328

b

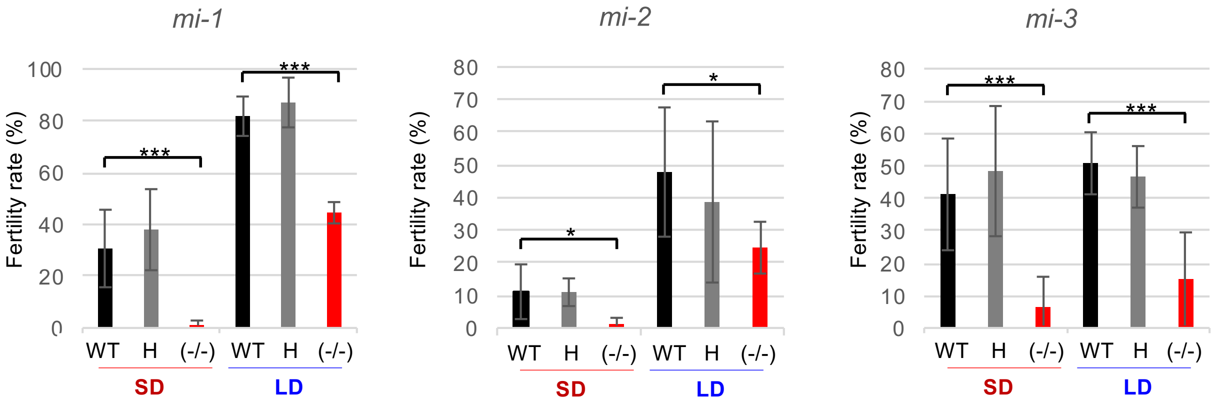

t-test, * *p*-value < 0.05, *** *p*-value < 0.001

Supplementary Figure 2. Segregation and fertility of *mir2118.*
a. Table showing the segregation rate of offspring from heterozygous *mi-1, mi-2,* and *mi-3.* The mutants were backcrossed once to Nipponbare, and the F2 populations were analyzed (BCF2). The deletion showed Mendelian inheritance in three *mir2118* lines using chi-square test. (WT: wild type from siblings, H: heterozygous, and -/-: homozygous). **b.** Fertility of *mir2118* under long day (LD) and short day (SD) conditions. Homozygous mutants (-/-) demonstrated sterility in *mi-1, mi-2,* and *mi-3*; wild-type (siblings) and heterozygotes of *mi-1, mi-2,* and *mi-3* set seeds under SD and LD conditions. Bar represents mean ± S.D. (N=3 or 4).* $p<0.05$, *** $p<0.001$, Student's *t*-test.

Supplementary Figure 3

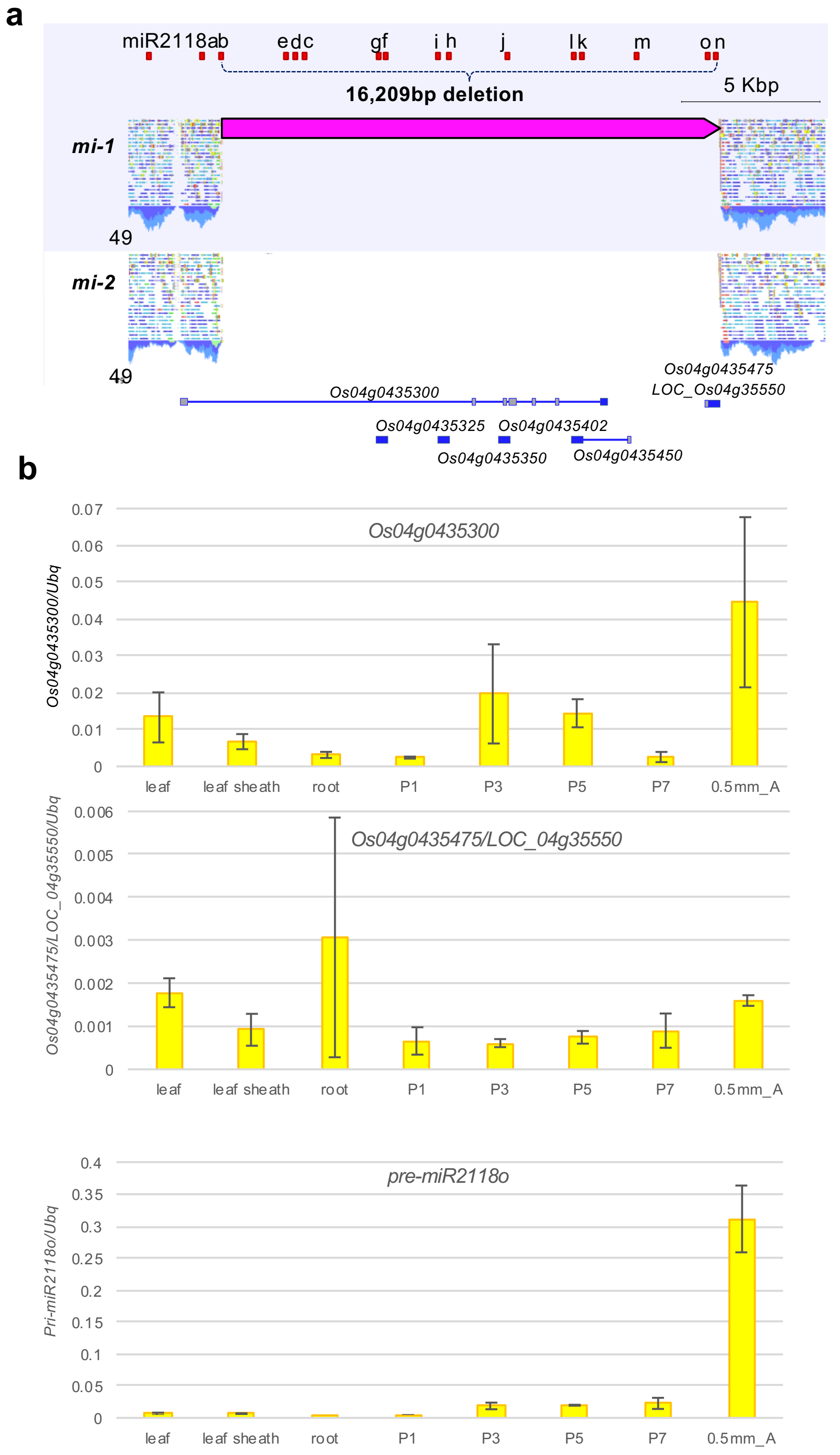

Supplementary Figure 3. Developmental expression of genes at miR2118-deleted regions. **a.** Schematic structure of miR2118 loci and genes predicted at the cluster in chromosome 4. **b.** qPCR analysis of two genes that were identified in RAP-DB and/or MSU, and *pre-miR2118o* at different developmental stages in wild-type. P1, P3, P5, and P7: 1cm, 3cm, 5cm and 7cm panicles, A: 0.5 mm anthers (Stage 2). Bar represents mean ± S.D. (N=3).

Supplementary Figure 4

a

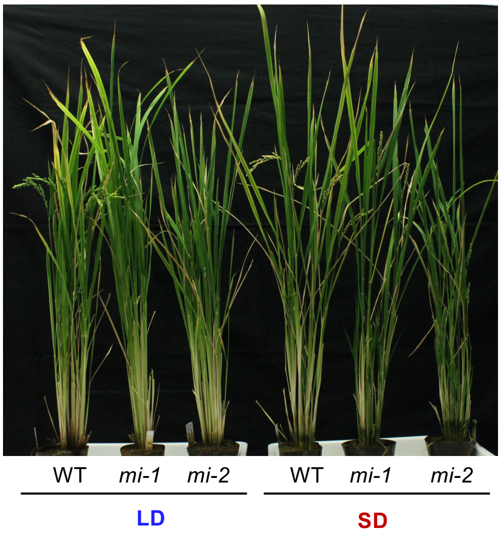

b

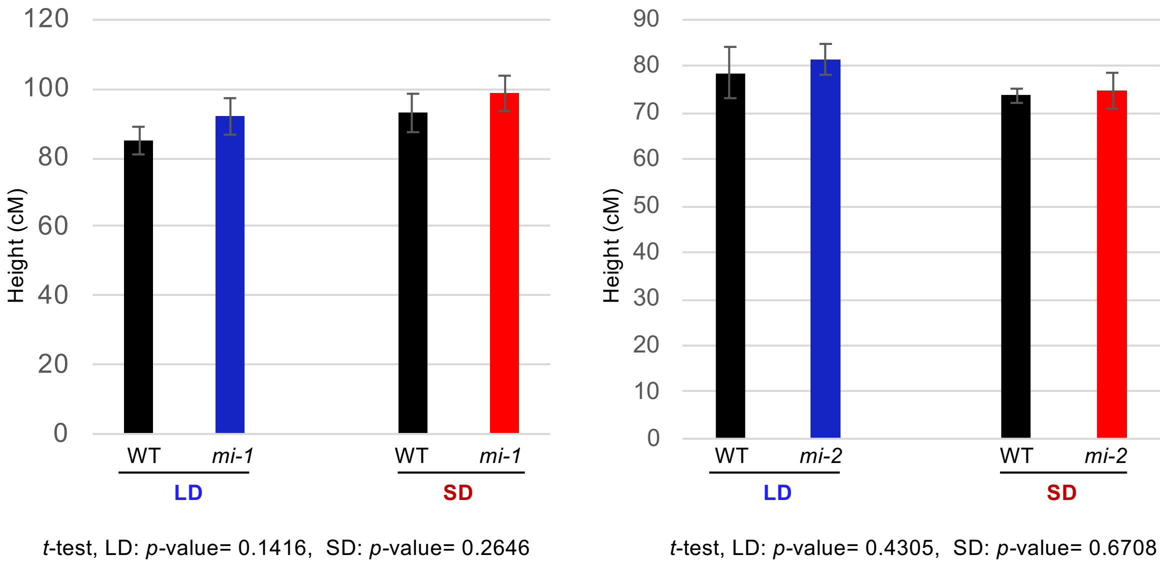

Supplementary Figure 4. Growth of *mir2118* under SD and LD conditions.
a. *mir2118* mutants grown under SD and LD conditions. **b.** Plant height of *mi-1* and *mi-2* in SD and LD (86 days). Each bar represents mean ± S.D. (N=3). No statistically significant differences were found between WT and *mi-1* or *mi-2* using Student's *t*-tests.

Supplementary Figure 5

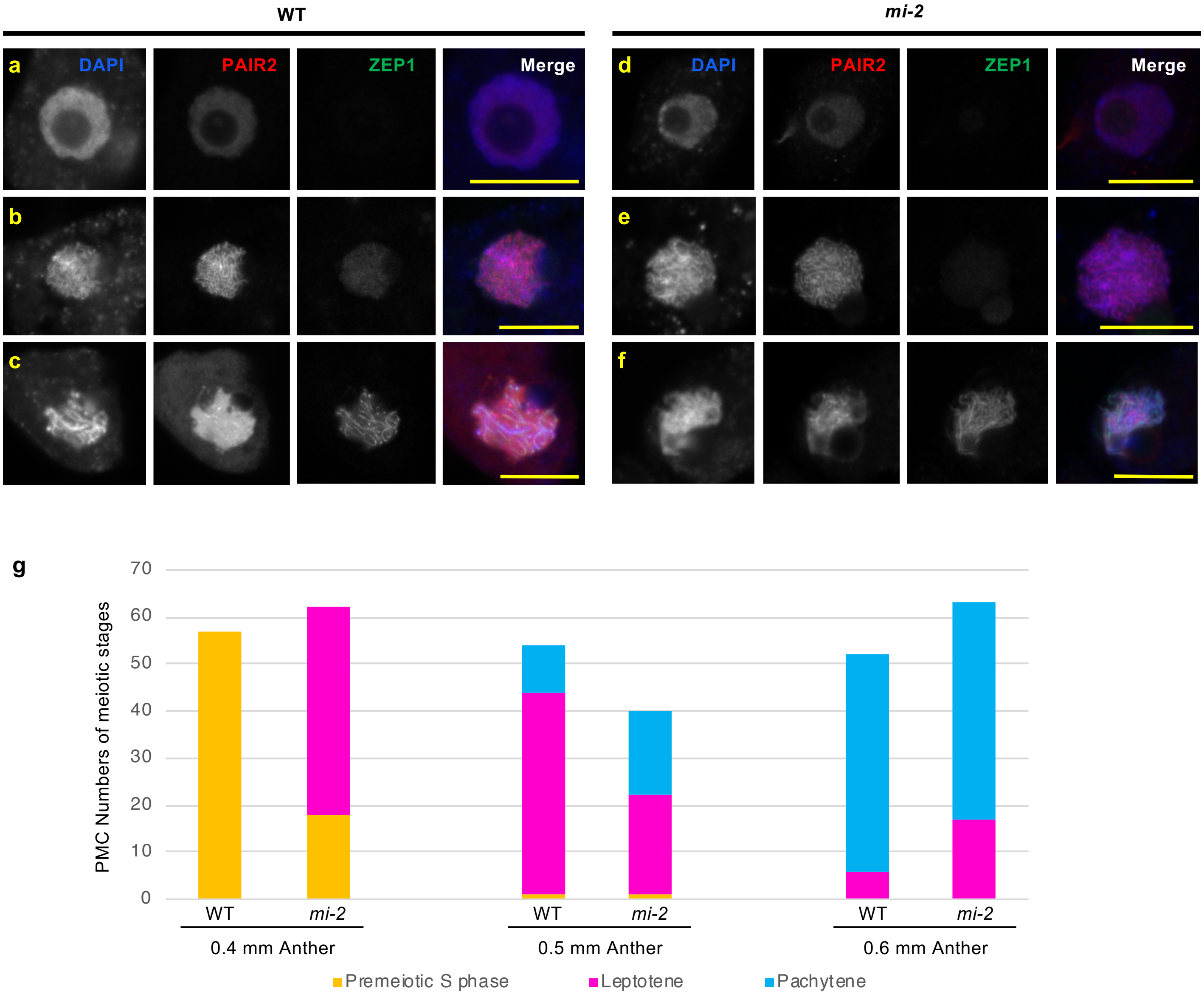

Supplementary Figure 5. Completion of homologous chromosome synapsis in PMC of *mir2118.*
a-f. Immuno-staining of pollen mother cells (PMC) at Stages 1 (**a, d**), Stage 2 (**b, e**), and Stage 3 (**c, f**) of WT (**a-c**) and *mi-2* (**d-f**) stained with PAIR2 (red) and ZEP1 (green) antibodies. DAPI staining is shown in blue. ZEP1 elongates along with homologous chromosome pairs. Yellow bars show 10μm.
g. Frequency of PMCs in the 0.4 mm to 0.6 mm anther at Stages 1 to 3.

Supplementary Figure 6

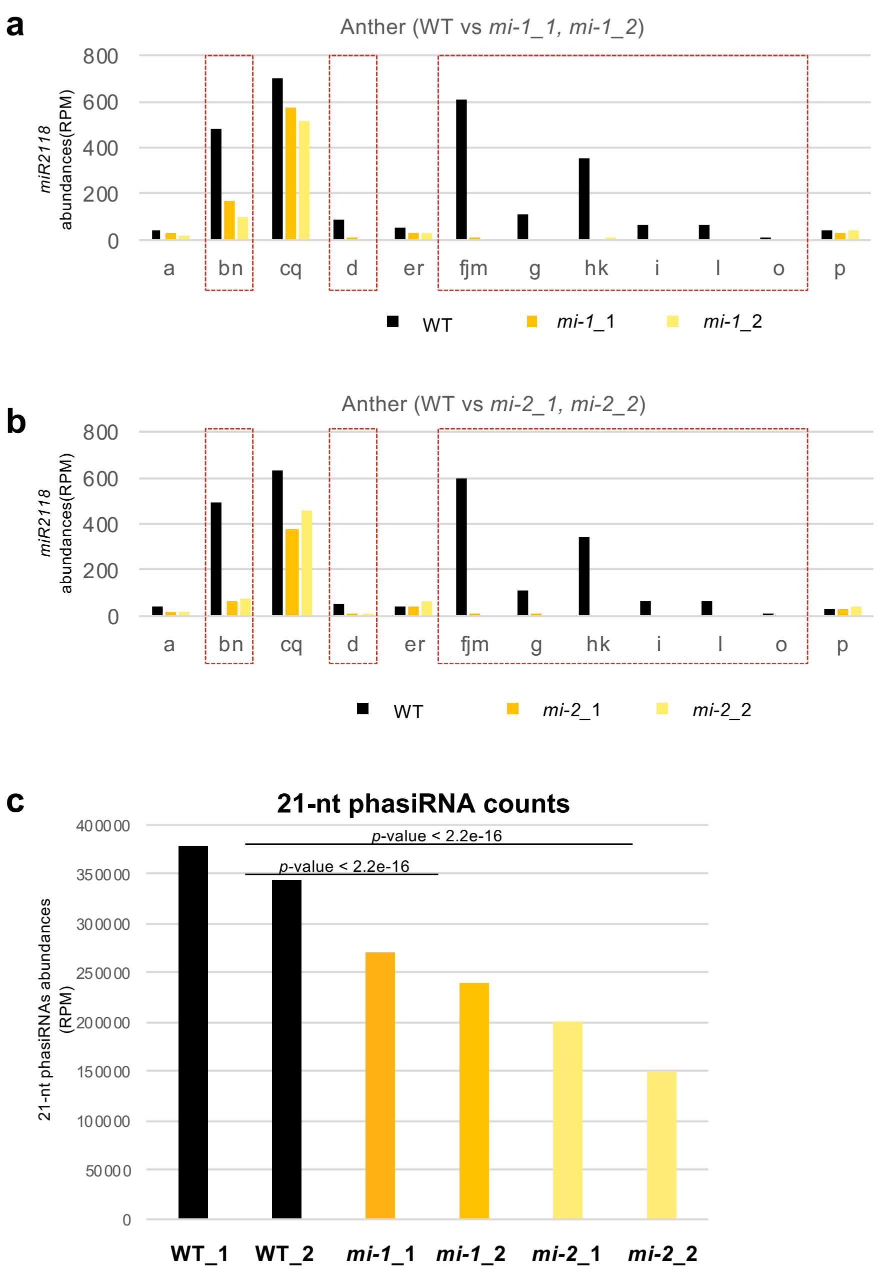

Supplementary Figure 6. Reduction of miR2118 expression in *mir2118.*
a and **b.** The expression levels of twelve mature miR2118s in WT, *mi-1,* and *mi-2,* as detected in small RNA-sequences. Expressions of eight miR2118bn, d, fjm, g, hk, i, l, and o were reduced in the 0.5 mm anthers of *mi-1* and *mi-2*. Small RNA-sequence was performed with two replicates for each sample. RPM; Reads per million. **c.** Read counts of 21-nt phasiRNA expressed in the anthers of the WT and *mir2118* mutants. The read counts demonstrated the two replicates of each sample. Statistically significant differences were found in anthers (Stage 2) between WT and *mi-1* or *mi-2* using chi-square test.

Supplementary Figure 7

a

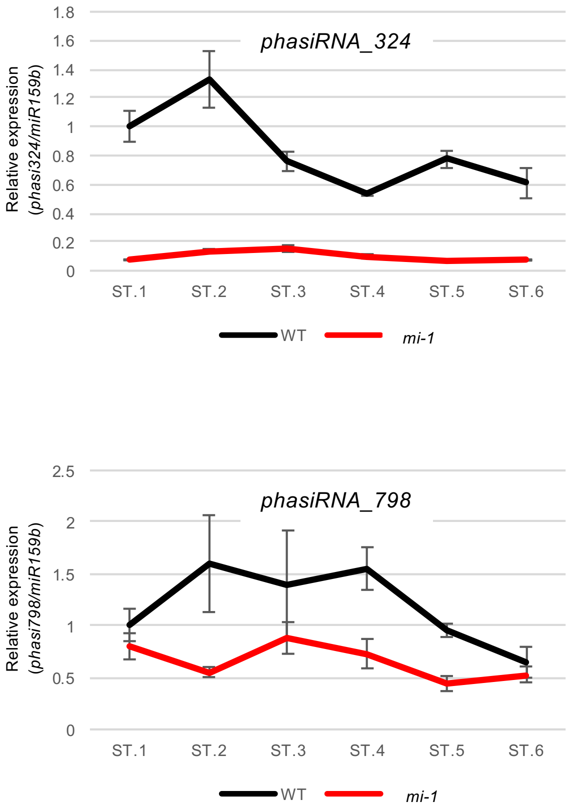

Supplementary Figure 7. qPCR analysis of two phasiRNAs at different anther developmental stages in WT and *mi-1.*

PhasiRNAs at *PHAS324* (**a**) and *PHAS798* (**b**) were decreased in *mi-1* during the anther development from Stage 1 to Stage 6. Data are mean ± S.D. of three technical replicates.

Supplemental Figure 8

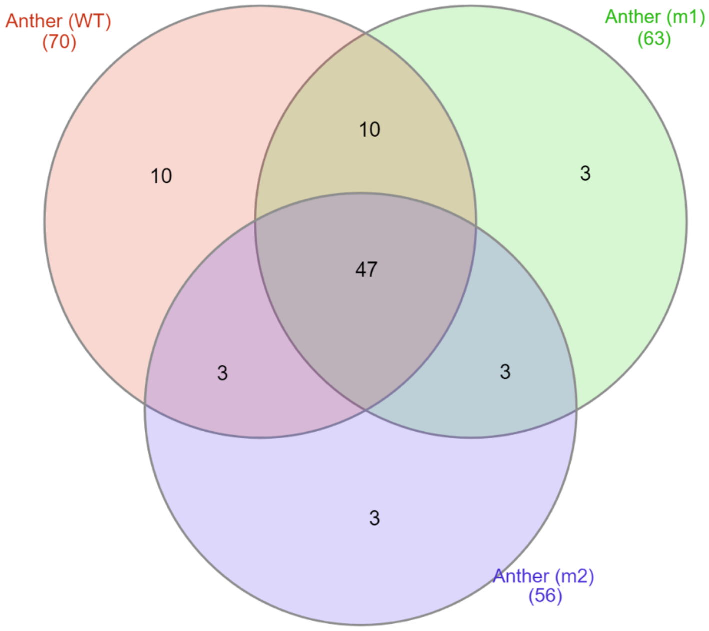

Supplementary Figure 8. 24-nt phasiRNA clusters in anther.
Venn-diagram showing overlapping 24-nt phasiRNA clusters in WT, *mi-1,* and *mi-2* in anther. Loss of miR2118 did not strongly affect the production of 24-nt phasiRNAs.

Supplementary Figure 9

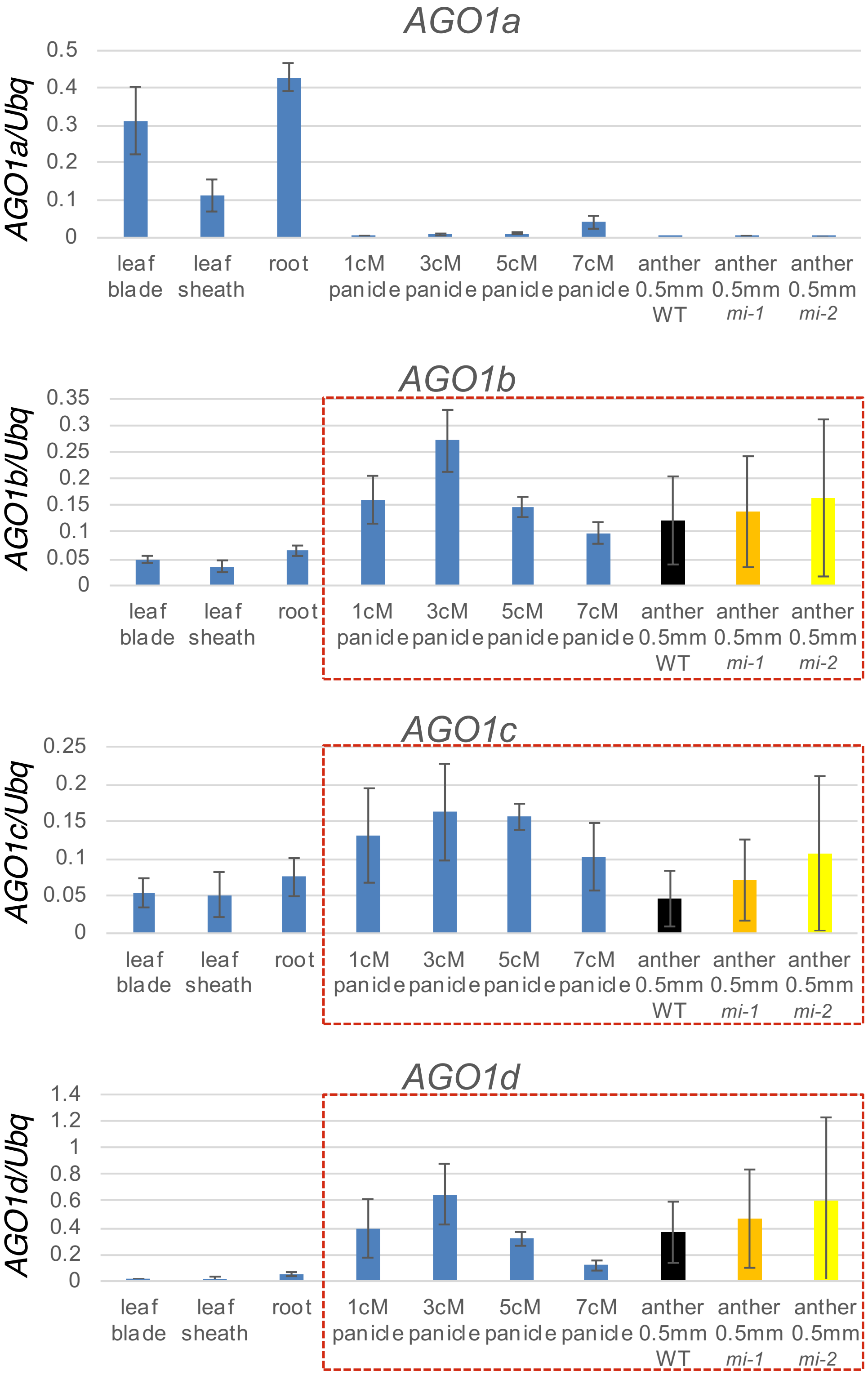

Supplementary Figure 9. Expression of *OsAGO1* subfamilies during vegetative and reproductive development.
qPCR analysis of *AGO1* genes in different rice tissues. No statistically significant differences were found in anthers (Stage 2) between WT and *mi-1* or *mi-2* using Student's *t*-tests. Data are mean ± S.D. of three biological replicates.

Supplementary Figure 10

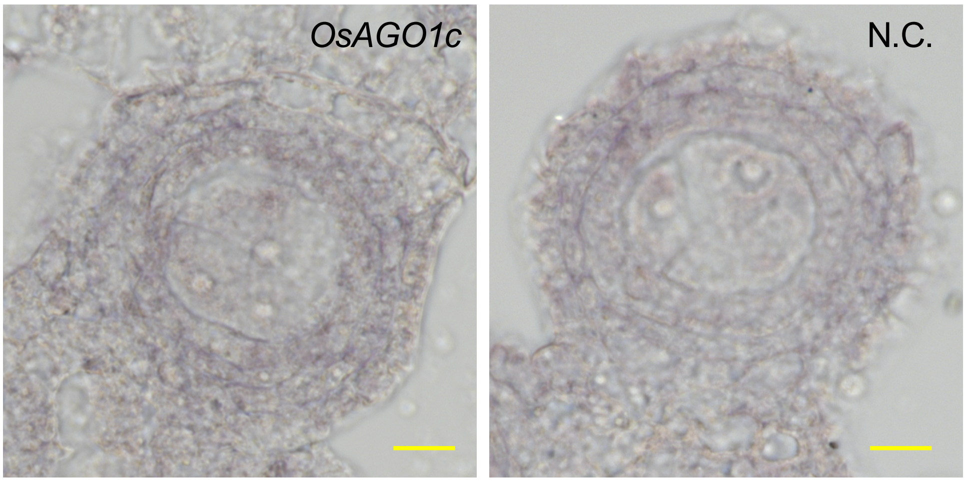

Supplementary Figure 10. *In situ* hybridization of Os*AGO1c* in the anther. *AGO1c* expression was not detected at Stage 1. Negative control (N.C.). Bars show 10µm.

Supplementary Figure 11

a

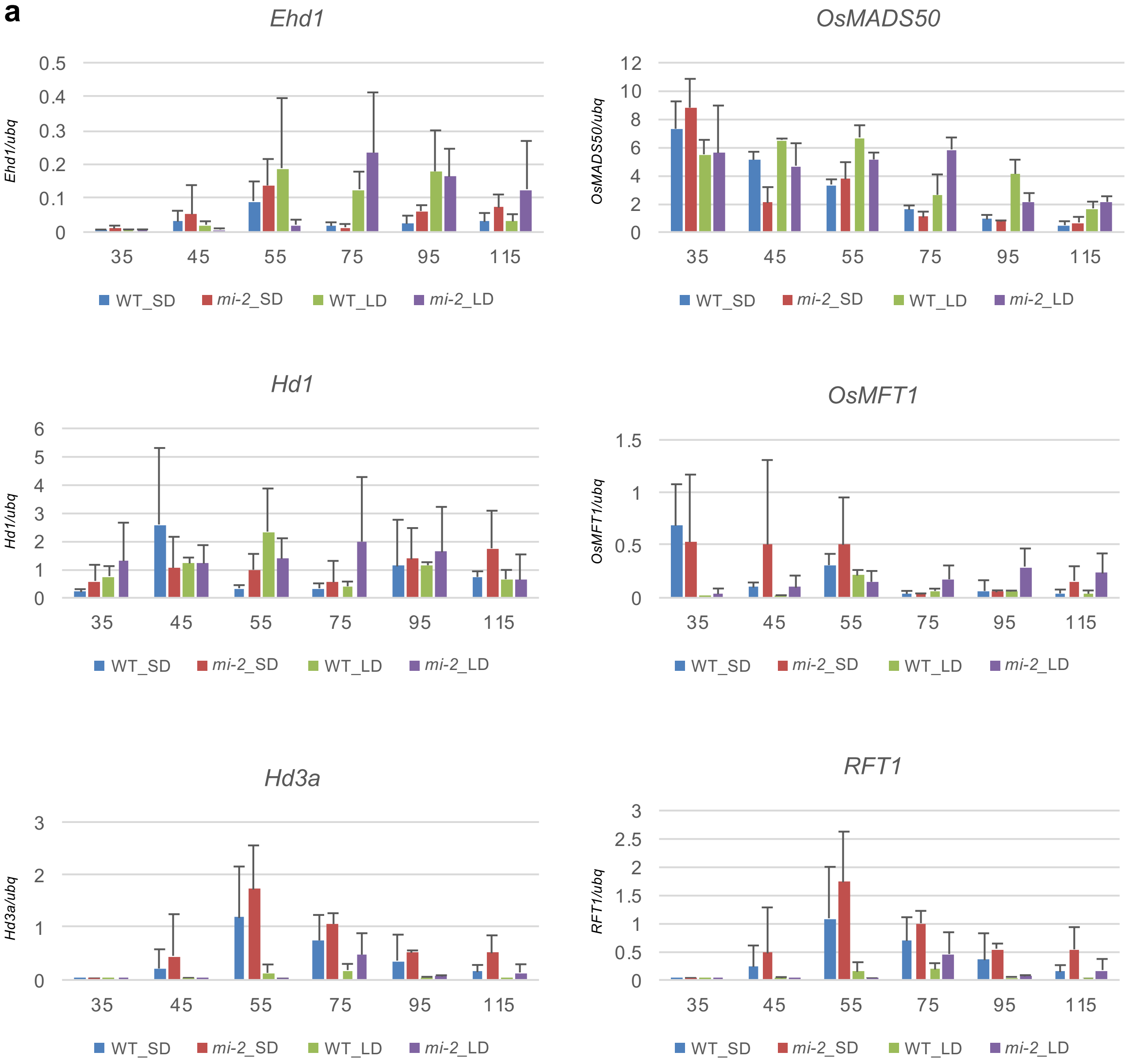

b

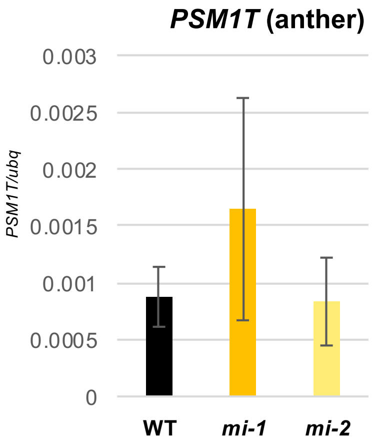

Supplementary Figure 11. Expression of photoperiodic genes in *mir2118* mutant.
a. Expression of *Ehd1, Hd1, Hd3a, OsMFT1, RFT1,* and *OsMDAS50* in *mi-2* and WT under SD and LD conditions. Expression levels of the flowering genes were not affected in *mi-2*. Leaves were collected from *mi-2* and WT plants at 35, 45, 55, 75, 95, and 115 days (ZT0) under short-day (SD) and long-day (LD) conditions. Data are mean ± S.D. of three biological replicates. **b.** Expression of *PSM1T* in 0.5mm anthers (at Stage 2) in WT, and *mir2118* plants under SD conditions.

Supplementary Table 1. Stigma numbers of WT, *mi-1* and *mi-2*

	Stigma numbers		
	2	3	4
WT	158	4	0
mi-1	115	16	5
mi-2	108	26	2

Fisher's Exact Test for Count Data

mi-1 , *p* -value = 9.182e-05

mi-2 , *p* -value = 4.066e-07

mir2118 mutants are written in *italic*

Supplementary Table 2. Pollination of WT pollen to *mir2118* floweres or *mir2118* polllen to WT flowers

P (♀)	×	P (♂)	flower numbers	fertile seeds	%
WT	×	WT	48	24	50
WT	×	WT	38	21	55
mi-3	×	WT	31	1	3 ***
mi-1	×	WT	48	4	8 ***
WT	×	*mi-3*	43	6	14 ***
WT	×	*mi-1*	34	2	6 ***

Binom test, *** p-value < 0.01

mir2118 mutants are written in italic

Supplementary Table 3. Summary of small RNAs reads mapped on the rice genome

	0.5mm anthers					
	WT_A1	WT_A2	*mi-1* _A1	*mi-1* _A2	*mi-2* _A1	*mi-2*_ A2
Input	9063663	14910428	5728785	6975046	8353559	4283352
mapped	5740964	9625588	3781495	4392294	4675912	2662278

mir2118 mutants are written in italic

Supplementary Table 4. Primer sequences used in this study

Primer	Sequences
**miR2118 gene targetting (GT)**	
miR2118 gRNA	CAATAGGAATGGGAGGCATC
**qPCR**	
Ubq -F	ATCAAGAACTAGAGCGTCACTC
Ubq -R	GCTGAAGCATCCAGCACAGTA
Os04g0435300 F	TATAGTTTGCTCTTCAGCCATGCGC
Os04g0435300 R	AGTACCTATATATACGCCTCTCACC
Os04g0435475 F	GCCATTGTTTGCACTTTTGCACTAC
Os04g0435475 R	TCTTGTCCTGAAACTCTGAAACCAG
WT_An_PHAS_324 qPCR F	tctagggttagcaacagcacagtg
WT_An_PHAS_324 qPCR R	ggcaaaagctagggactggttg
WT_An_PHAS_1586 qPCR F	gccattgccatgtttgttggactttg
WT_An_PHAS_1586 qPCR R	ctgcaatgtgacatggaggtgaatc
AGO1a qPCR F	GAAAGTTTCCACTCTGTCTGCGGG
AGO1a qPCR R	AGTAGTACCCGTTGTCCAACTGG
AGO1b qPCR F	AGCACCGGTGAGAGTTCTGGAG
AGO1b qPCR R	CACCTGGATGGTGTGAACCTTG
AGO1c qPCR F	AGCTGCAGCTGCAACTCTCCATC
AGO1c qPCR R	TTCCACCAGGATCTGGAGCCT
AGO1d qPCR F	AGGAGCACTGCATTACTGGTAGTG
AGO1d qPCR R	GATTCAGCTCACATCCAGACGAAC
OsMFT1 qPCR F	GACCTAGCTAGCTAATAAGCCATC
OsMFT1 qPCR R	CCACATAAACGACGACATGATATGG
OsMADS50 -F	CAGGCCAGGAATAAGCTGGAT
OsMADS50 -R	TTAGGATGGTTTGGTGTCATTGC
Ehd1 -F	TGCAAATGGCGCTTTTGAT
Ehd1 -R	ATATGTGCTGCCAAATGTTGCT
RFT1-F	TGACCTAGATTCAAAGTCTAATCCTT
RFT1-R	TGCCGGCCATGTCAAATTAATAAC
Hd3a -F	GCTCACTATCATCATCCAGCATG
Hd3a -R	CCTTGCTCAGCTATTTAATTGCATAA
Hd1-F	TCAGCAACAGCATATCTTTCTCATCA
Hd1 -R	TCTGGAATTTGGCATATCTATCACC
PSM1T qPCR F	caaatactggaacacagtcatcag
PSM1T qPCR R	catggacaattggtgataccaggc
**small RNA qPCR**	
miR159b F qPCR	tttggattgaagggagctctg
phasi324 F qPCR	CTGTGCTGTTGCTAACCCTAG
phasi798 F qPCR	TCATAGACTGTGTAAGCTGCT
ISH probe	
AGO1b ISH F	AGCACCGGTGAGAGTTCTGGAG
AGO1b ISH R	GCTGAAACTGTTGTTGGACTTGTCC
AGO1c ISH F	AGCTGCAGCTGCAACTCTCCATC
AGO1c ISH R	GGCATCCACAGGTTGTTCCTGAG
AGO1d ISH F	AGGAGCACTGCATTACTGGTAGTG
AGO1d ISH R	GCTCATGTGGAGCATCCATTGCTTG
MEL1 ISH F	GCATTGTCTCAAGCAGAGTTAAGGC
MEL1 ISH R	CCTGAAATCACCAAATACCG
osa-miR2118fjm	5DiGN/TAGGAATGGGAGGCATCAGGAA/3DiGN
miRCURY LNA (negative control)	5DiGN/GTGTAACACGTCTATACGCCCA/3DiGN

Gene name and small RNAs are written in *italic*
