# Supplementary material for: miR2118-dependent U-rich phasiRNA production in rice anther wall development
Source: Nat Commun. 2020 Jun 19;11:3115. doi: 10.1038/s41467-020-16637-3 (PMC7305157; doi:10.1038/s41467-020-16637-3)
Supplement: Supplementary file 3 — Description of Additional Supplementary Files [file 41467_2020_16637_MOESM3_ESM.docx]

**Descriptions of Additional Supplementary Files**

**File name:** Supplementary Movie 1

**Description:** 3D structure of 0.5 mm anthers in WT. Anthers were visualized using Lightsheet microscopy by detecting autofluorescence over 585 nm.

**File name:** Supplementary Movie 2

**Description:** 3D structure of 0.5 mm anthers in *mi-1*. Anthers were visualized using Lightsheet microscopy by detecting autofluorescence over 585 nm.

**File name:** Supplementary Movie 3

**Description:** 3D structure of 0.5 mm anthers in *mi-2*. Anthers were visualized using Lightsheet microscopy by detecting autofluorescence over 585 nm.

**File name:** Supplementary Data 1

**Description:** The genomic position of 21-nt phasiRNA clusters in anthers.

**File name:** Supplementary Data 2

**Description:** Differentially expressed proteins (<0.8 fold change) in *mi-1* mutants (p-value<0.05)
