# Supplementary material for: miR2118-dependent U-rich phasiRNA production in rice anther wall development
Source: Nat Commun. 2020 Jun 19;11:3115. doi: 10.1038/s41467-020-16637-3 (PMC7305157; doi:10.1038/s41467-020-16637-3)
Supplement: Supplementary file 9 — Reporting Summary [file 41467_2020_16637_MOESM9_ESM.pdf]

nature research

Corresponding author(s): Reina Komiya

Last updated by author(s): May 8, 2020

Reporting Summary

Nature Research wishes to improve the reproducibility of the work that we publish. This form provides structure for consistency and transparency in reporting. For further information on Nature Research policies, see Authors & Referees and the Editorial Policy Checklist .

Statistics

For all statistical analyses, confirm that the following items are present in the figure legend, table legend, main text, or Methods section.

n/a	Confirmed	
☐	☒	The exact sample size (*n*) for each experimental group/condition, given as a discrete number and unit of measurement
☐	☒	A statement on whether measurements were taken from distinct samples or whether the same sample was measured repeatedly
☐	☒	The statistical test(s) used AND whether they are one- or two-sided<br>*Only common tests should be described solely by name; describe more complex techniques in the Methods section.*
☒	☐	A description of all covariates tested
☒	☐	A description of any assumptions or corrections, such as tests of normality and adjustment for multiple comparisons
☐	☒	A full description of the statistical parameters including central tendency (e.g. means) or other basic estimates (e.g. regression coefficient) AND variation (e.g. standard deviation) or associated estimates of uncertainty (e.g. confidence intervals)
☐	☒	For null hypothesis testing, the test statistic (e.g. *F*, *t*, *r*) with confidence intervals, effect sizes, degrees of freedom and *P* value noted<br>*Give P values as exact values whenever suitable.*
☒	☐	For Bayesian analysis, information on the choice of priors and Markov chain Monte Carlo settings
☒	☐	For hierarchical and complex designs, identification of the appropriate level for tests and full reporting of outcomes
☒	☐	Estimates of effect sizes (e.g. Cohen's *d*, Pearson's *r*), indicating how they were calculated

Our web collection on statistics for biologists contains articles on many of the points above.

Software and code

Policy information about availability of computer code

Data collection: n/a

Data analysis:

- Trimmomatic-0.38
- tophat/2.1.1
- samtools/1.3.1
- PHASIS/3.3
- bowtie/1.0.0 (used by PHASIS)
- R/3.5.2
- ggseqlogo/0.1
- ggplot2/3.2.1
- IGV/2.5.2
- CLC/11.0.1
- ZEN2011

Microscopy
1.CCarl Zeiss Lightsheet Z.1
Detection lens: 20x PlanApochomat,
Illumination lens: 10x LSFM Clearing

2. Nikon NiE
DS-Ri2 Color Camera
40x/0.95 Plan Apoλ lens

3. Carl Zeiss LMS780 Observer.Z1
20x/0.80 Alan Apo, 40x/1.40 Plan Apo, 63x/1.46 αPlan Apo,

For manuscripts utilizing custom algorithms or software that are central to the research but not yet described in published literature, software must be made available to editors/reviewers. We strongly encourage code deposition in a community repository (e.g. GitHub). See the Nature Research guidelines for submitting code & software for further information.

Data

Policy information about availability of data

All manuscripts must include a data availability statement. This statement should provide the following information, where applicable:

- Accession codes, unique identifiers, or web links for publicly available datasets
- A list of figures that have associated raw data
- A description of any restrictions on data availability

Whole-genome sequencing data and small RNA transcriptome data have been deposited in the DNA Bank of Japan (DDBJ), under the accession codes PSUB010670 (whole genome sequences) and PSUB010669 (small RNA transcriptome). Proteome data were deposited to JPOST, under the accession code PXD015440.
The identification is described in the data and code avairability section.

Field-specific reporting

Please select the one below that is the best fit for your research. If you are not sure, read the appropriate sections before making your selection.

☒ Life sciences ☐ Behavioural & social sciences ☐ Ecological, evolutionary & environmental sciences

For a reference copy of the document with all sections, see nature.com/documents/nr-reporting-summary-flat.pdf

Life sciences study design

All studies must disclose on these points even when the disclosure is negative.

Sample size	No sample size calculation was performed. Sample size was chosen according to the standard in the plant biology field.
Data exclusions	n/a
Replication	All the experiments were reproducible in the repeated experiments.
Randomization	Plant samples were placed randomly in the plant facility.
Blinding	No blinding was applied for sampling.

Reporting for specific materials, systems and methods

We require information from authors about some types of materials, experimental systems and methods used in many studies. Here, indicate whether each material, system or method listed is relevant to your study. If you are not sure if a list item applies to your research, read the appropriate section before selecting a response.

Materials & experimental systems

n/a	Involved in the study	
☐	☒	Antibodies
☒	☐	Eukaryotic cell lines
☒	☐	Palaeontology
☒	☐	Animals and other organisms
☒	☐	Human research participants
☒	☐	Clinical data

Methods

n/a	Involved in the study	
☒	☐	ChIP-seq
☒	☐	Flow cytometry
☒	☐	MRI-based neuroimaging

Antibodies

Antibodies used

(1) MEL1 (Komiya et al., 2014)
Two oligopeptides, MEL1-N (CVYGAPMPAAHHQGAYQ) and MEL1-C (GQAVAREGPVEVRQLPKC) were used to induce antibody production in rabbits. The antiserum was affinity purified. To confirm that the IP fraction contained MEL1 proteins, MEL1 proteins (around 120 kD) were sequenced by liquid chromatography-mass spectrometry, HCTultra (Bruker) (Komiya et ai., 2014).

(2) PAIR2 (Nonomura et al., 2006)

The entire coding region of PAIR2 cDNA (DDBJ Accession No. AB109238) (Nonomura et al., 2004b) was amplified by PCR using primers P609 (5′-CACCATGGTGATGGCTCAGAAGACGAAG-3′) and P610 (5′-TCACTGAACTTGAACTTGAACTTGGGAC-3′). The PCR product was cloned into the pENTR-TOPO plasmid and re-inserted into pDEST17 with a 6× histidine (6×His) repeat at the 5′-end of the multiple cloning site using the Gateway system (Invitrogen). After transformation of Escherichia coli strain BL21-AI (Invitrogen) with the plasmid, expression of the fusion protein was induced by adding L-arabinose to a final concentration of 0.2% (w/v) in LB liquid culture.

The recombinant peptides expressed were extracted 4 or 6 hours after induction using the BugBuster protein extraction reagent (Novagen). The 6×His-tagged peptides in the soluble fraction were purified using a HisTrap kit (from Amersham Biosciences), respectively, according to the manufacturer's instructions. 250 μg of PAIR2 recombinant peptides were injected into a rabbit and guinea pig every two weeks, respectively. Immune sera were extracted 52 days after the first injection.

(3) ZEP1 (Liu et al.,2016)

To generate the antibody against ZEP1, an N-terminal region of OsZEP1 cDNA encoding the 17-436 aa region was amplified by PCR with a primer pair of 5'-CACCTTAGAAGTACTGTTTCAGGGCCCGTCTCTCGCTGGATCCACC-3' and 5'-TCATTCAGCAGATCTAGAATCCTCC-3'. The amplicon was cloned into pENTR/D-TOPO, reinserted into pDEST17 (Gateway system, Invitrogen), and transferred into E. coli strain BL21-AI (Invitrogen). 6xHis tagged OsZEP1 peptide was purified using HisTrapFF crude kit (GE Healthcare) according to manufacture's instruction. The purified peptide was immunized to rat, and the antiserum was used for immunostaining.

(4) Secondary antibody

Alexa Fluor 488-conjugated anti-rabbit IgG (Invitrogen, A-11034)

Alexa Fluor 647-conjugated anti-rat IgG (Invitrogen, A-21247)

Validation

(1) MEL1 (Komiya et al., 2014)

No anti-MEL1 signal was obtained in mel1 mutnats. Furthermore, MEL1 proteins (around 120 kD) which were immnopurified with anti-MEL1, were sequenced by liquid chromatography-mass spectrometry.

(2) PAIR2 (Nonomura et al., 2006)

This antibody is conformed by western blotting (optimal size) and immunostaning of pollen mother cells as a leptotene marker.

(3) ZEP1

This antibody is conformed by immunostaning of pollen mother cells as a zygotene and pachytene marker.
